# A qualitative inquiry of rural-urban inequalities in the distribution and retention of healthcare workers in southern Nigeria

**DOI:** 10.1371/journal.pone.0266159

**Published:** 2022-03-29

**Authors:** Ogonna N. O. Nwankwo, Chukwuebuka I. Ugwu, Grace I. Nwankwo, Michael A. Akpoke, Collins Anyigor, Uzoma Obi-Nwankwo, Sunday Andrew, Kelechukwu Nwogu, Neil Spicer

**Affiliations:** 1 London School of Hygiene and Tropical Medicine, London, United Kingdom; 2 World Health Organization Field Office, Awka, Nigeria; 3 Department of Paediatrics, University of Calabar Teaching Hospital, Calabar, Nigeria; 4 Ola During Childrens Hospital, Freetown, Sierra Leone; 5 Department of Family Medicine, Federal Medical Center, Abuja, Nigeria; 6 Detar/A&M University FM Residency Program, Victoria, Texas, United States of America; 7 Christian Medical and Dental Association, National Office, Abuja, Nigeria; 8 Unicef Country Office, Abuja, Nigeria; 9 Department of Global Health and Development, Faculty of Public Health and Policy, London School of Hygiene and Tropical Medicine, London, United Kingdom; University of Tennessee Health Science Center College of Pharmacy Memphis, UNITED STATES

## Abstract

**Introduction:**

Globally, the population in rural communities are disproportionately cared for by only 25% and 38% of the total physicians and nursing staff, respectively; hence, the poor health outcomes in these communities. This condition is worse in Nigeria by the critical shortage of skilled healthcare workforce. This study aimed to explore factors responsible for the uneven distribution of healthcare workers (physicians and nurses) to rural areas of Ebonyi State, Nigeria.

**Methods:**

Qualitative data were obtained using semi-structured in-depth interviews and focus group discussions from purposively selected physicians, nurses, and policymakers in the state. Data was analysed for themes related to factors influencing the mal-distribution of healthcare workers (physicians and nurses) to rural areas. The qualitative analysis involved the use of both inductive and deductive reasoning in an iterative manner.

**Results:**

This study showed that there were diverse reasons for the uneven distribution of skilled healthcare workers in Ebonyi State. This was broadly classified into three themes; socio-cultural, healthcare system, and personal healthcare workers’ intrinsic factors. The socio-cultural factors include symbolic capital and stigma while healthcare system and governance issues include poor human resources for health policy and planning, work resources and environment, decentralization, salary differences, skewed distribution of tertiary health facilities to urban area and political interference. The intrinsic healthcare workers’ factors include career progression and prospect, negative effect on family life, personal characteristics and background, isolation, personal perceptions and beliefs.

**Conclusions:**

There may be a need to implement both non-financial and financial actions to encourage more urban to rural migration of healthcare workers (physicians and nurses) and to provide incentives for the retention of rural-based health workers.

## Introduction

Healthcare workers form a key component of the health system necessary for achieving its primary goal of enhancing the quality of health of the population [1]. Physicians and nurses/midwives have been identified by the World Health Organisation (WHO) as key predictors of good health outcomes and as a measure for assessing the quality of care for every population [1, 2]. The ratio of physicians and nurses/midwives (hereafter referred to as ’healthcare workers’ (HCWs) to the population had been shown to be a consistent predictor of failure of countries not meeting the Millennium Development Goals (MDGs) [1].

The current global shortage of HCWs is forecasted to persist in the foreseeable future with a projected need for 45 million physicians and nurses/midwives by 2030 [3]. Worse hit are low- and middle-income countries (LMICs), including Nigeria, which suffer from a critical shortage of HCWs [1, 4]. This shortage has been made worse by the migration of available HCWs from such countries to more developed countries [5, 6]. In addition to this, there is an inequitable distribution of the available HCWs between different regions and even between rural and urban areas in these countries [7]. Globally, it is estimated that rural dwellers constitute a significant proportion of the population, but they are disproportionately cared for by 25% and 38% of the total physicians and nursing workforce respectively [1, 8]. This is a problem that affects almost all countries worldwide [9, 10].

In Nigeria, rural community residents, despite making up about one-half of the population, have access to only an estimated 12% and 19% of the total number of physicians and nurses, respectively [11, 12]. This inequitable distribution has resulted in significantly poorer indices in all indicators of health for rural dwellers compared to their urban counterparts. Thus, national surveys in Nigeria consistently show that rural community residents are far less likely to receive antenatal care from a skilled provider (doctor or nurses/midwife inclusive) compared to urban dwellers [13, 14]. Also, only about 28.0% of the deliveries in rural areas were attended to by a skilled birth attendant compared to 67.6% in urban areas [14]. This deficit in maternal and child care has contributed to the disproportionate worse maternal and infant mortality rates seen in rural areas compared to the urban areas [13]. Furthermore, rural residents are also exposed to higher costs while seeking better care in urban areas due to a loss of confidence in public health facilities in rural areas [15]. All of this situation had been identified as one of the main hurdles against Nigeria’s past efforts to meet most of its health-related MDGs with the potential risk of derailing her efforts towards meeting the health-related Sustainable Developmental Goals (SDGs) in 2030 [16].

According to the WHO, understanding the contextual issues driving the distribution of HCWs has been identified as key to enabling a proper framing and implementation of bespoke strategies for the attraction, recruitment and retention of HCWs in rural areas [2, 17]. However, country differences exist and hence the repeated calls by the WHO and other authors to make available more evidence on these local contextual factors to enable the framing of solutions fittingly tailored for each region [2, 17, 18]. Although Nigeria is one of the countries with a critical shortage of HCWs in rural areas, the evidence is thin on context-specific drivers that have influenced this inequitable distribution, necessary for crafting actionable policies [19]. The few available evidence seen is highly reliant on using quantitative methodologies in addressing the issue [19–21]. Thus, this qualitative study is appropriate for such areas of research in eliciting contextual issues around urban-rural inequitable distribution of the health workforce [22]. Many factors have been reported to be the reasons why most HCWs would rather not work or be retained in rural areas. These are described as ’the pull-push or stick -stay factors’ [9, 10]. However, most studies have overlooked exploring the issues from the perspectives of urban health workers (especially those with past experience of working in rural areas) and policymakers which can give insights into the factors discouraging urban HCWs from relocating to or being retained in rural areas. Evidence in such areas according to the World Health Organisation are needed areas of research in attempting to address the inequitable distribution of health workers to rural and remote areas [23]. Our findings would be useful to health policymakers in the study setting as well as other similar settings around the world. The work is limited to exploring the issues affecting only physicians and nurses. Thus, this study aims to determine the contextual factors responsible for the current inequitable distribution of healthcare workers (physicians and nurses) between the rural and urban areas in Ebonyi State.

## Methods

### Study setting

This study was focused on Ebonyi State, Nigeria. It is located in the south-eastern part of Nigeria and also is one of the 36 states that make up Nigeria. It has 13 Local Government Areas (LGAs), with Abakaliki, Ebonyi, and Afikpo North LGAs being classified as urban while the rest (Onicha, Ohaozara, Ivo, Ishielu, Ezza-North, Ezza- South, Afikpo-South, Ohaukwu, Ikwo, and Izzi LGAs) are classified as rural. The state has a population of 2,176,977 at the last census count and the main tribe is Igbo [24]. The average population density is 286 persons per square km. Nigeria currently runs a three-tier form of government and of which the healthcare provision and funding follows the tiers–Federal, State, and Local Governments [25]. The Federal Government is mostly responsible for the provision of tertiary care while the State and the Local Governments largely takes care of the secondary and primary healthcare, respectively. Usually, the Primary Health Care (PHC) facilities and a majority of the secondary level facilities are responsible for the healthcare needs of the rural community residents. Nigeria has a very low density of highly skilled health workers (physicians and nurses) estimated at 38.9 physicians and 148 nurses per 100,000 persons [26]. These fall below the WHO recommendation, with most HCWs working in the urban area [26].

### Study population

Urban-based highly skilled HCWs composed of only physicians and nurses of different cadres and years of experience were mainly recruited for the study. Also, some rural-based HCWs and policymakers within the State Ministry of Health (SMoH) were recruited. A significant number of all the urban based participants (17 out of the 18) had worked in a rural area before moving to an urban area.

### Sampling strategy

Overall, for the interviews, twenty-three key informants comprising thirteen physicians, six nurses and four health policymakers were purposively selected (Table 1). The aim was to maximize the diversity and variability in the data. Although qualitative sampling does not aim to achieve representativeness, a good mix of participants will yield better-nuanced data in interviews [27]. Seven urban-based HCWs made up of three physicians and four nurses were selected using purposive and snowballing method for the focus group discussion (FGD) that held.

**Table 1:** Characteristics of selected participants for the IDIs.

Participants Characateristics	Urban-based	Rural-based
1.Early career physicians (0–10 yrs)	6 (4 males, 2 females)	1 (1 male)
2.Mid to late career physicians (>10yrs)	3 (1 male, 2 female)	3 (3 males)
3.Early career murses (0–10 yrs)	3 (3 females)	1 (female)
4.Mid- to late-career nurses (>10yrs)	2 (2 females)	
5.Policy makers	4 (2 males, 2 females)	
Sub- total	18	5
**Overall total**	23	

### Inclusion and exclusion criteria

All the selection in both the interviews and FGD was based on participants’ working experience, previous work in rural areas and socio-demographic variables deemed contributory to participants’ insight on the subject matter such as marital status, age, stage in career, and place of origin. These are variables that have been shown in past studies to influence one’s choice of residing and working in a rural area [2, 17]. In recruiting the participants into the study, this characteristics were looked out for to ensure we get some contrasting diversity in the participants characteristics in these areas. Healthcare students were excluded from being part of the study as we wanted the work to represent the opinions of those already working in the health system who have made choices on working in any of the areas.

The majority of the urban-based healthcare workers had worked and lived in rural areas while most of the rural-based health workers had worked and lived in urban areas prior to working in rural areas. Data saturation was reached by the time we had conducted the 23 interviews and one group discussion as participants were no longer providing any new insights. There were no repeat interviews or group discussions.

### Data collection procedures

The topic guides were developed from literature reviews, expert opinions and personal experience of the researchers as seen in S1 and S2 Files. The tools were pre-tested with similar cadres of health workers like our participants, with few refinements made following the pre-test. A semi-structured topic guide was used as it was considered appropriate to elicit in-depth data from the participants while allowing iterative probing by the researcher [28]. The guide contained questions attempting to address the objectives of the study in sections, while prompts for each question were drawn from literature reviews.

Prior contact and introduction to the study were made using phone calls or contact meetings, and individual scheduling for the interviews was made. All the HCWs contacted were willing to participate. On the scheduled day of each interview, each participant was given the information sheet and was requested to give written informed consent if they still wished to participate after reading. A snowballing approach was used in recruiting participants for the FGD where we initially purposively recruited a doctor and nurse who thereafter helped recruit other HCWs who met our criteria for the FGD.

The interviews were conducted in quiet spaces (usually in private offices or the homes of the participants) as chosen by the participant and deemed fit for purpose considering the need to minimize the power dynamics between the researcher and the interviewee [27, 28]. The interviews were conducted primarily by ONON. The interviews explored participants’ experiences around working in rural areas and their understanding of factors that influence HCWs’ decisions to work in such areas.

The participants of the FGD included four doctors and three nurses working in public-based health facilities who have all had the experience of working in both urban and rural health facilities. The FGD was held in one of the clinic offices immediately after the clinic sessions with a moderator and a note-taker. The participants all signed the consent forms and verbally agreed not to divulge the comments of other individuals within the discussion. A brief moment of personal debrief and reflection was observed following the FGD. This provided an opportunity to make additional notes on key non-verbal cues and other behavioural expressions deemed contributory to the discourse. It also helped to reflect on the emerging themes and prepare for the analysis.

All the interviews and the focus group discussion were conducted in English and were audio-recorded with each lasting between 30 to 45 minutes. The focus group discussion lasted for about one hour.

### Conceptual framework

Our analysis was informed by the ‘the pull, push, stick and stay factors’framework which is used to explore reasons for migration amongst health workers internally or externally [9, 19]. Lots of factors have been demonstrated to be the reasons why most HCWs would rather not work in rural areas, described as ‘the pull, push, stick and stay factors’. The ‘push’ factors are those that would make a health worker to decide to leave his employment in a rural area and move to an urban area while ‘pull’ factors are responsible for migration in the opposite direction. The ‘stay’ factors are those reasons that would make urban workers, irrespective of the ‘pull’ factors to decide to continue working in the urban area whereas the ‘stick’ factors are reasons that would make them refuse to leave from the rural area when employed. Our analysis considered more of the stay and stick factors.

### Data handling and analysis

The interviews were manually transcribed and subsequently the transcripts were read through to help in familiarising with the data. The analysis involved the use of both inductive and deductive reasoning in an interactive way to achieve data reduction, organisation, clarification, and evaluation. The deductive phase allowed the comparison of the data against predefined generalizations and themes from already existing theories. This approach of combining both the inductive and deductive methods is argued to improve interpretive understanding of the research data [22].

The process began with initial familiarisation with the data by reading the transcripts several times over. Thereafter, recurrent ideas within the data were detected and labelled as codes manually. This generated a list of codes observed to be recurring commonly across the earlier interviews. The next phase was the grouping of all the codes which aimed to identify similar underlying ideas both within each case and across the cases. This helped to reduce the codes further into groups from which the themes were abstracted. For this research, we classified a theme as a concept that expresses something significant about the data with respect to the research question and also represents a patterned meaning within the data [22, 27, 28]. While the initial analysis was conducted by the first author, ONON, the premilinary codes and themes were reviewed by one of the co-authors, CIU, regarding the transcripts. Subsequently, the other co-authors reviewed the final codes and themes that was derived and helped in further interpretation of the underlying data.

Ultimately, themes were then abstracted from the categories of codes. Some of the themes related literally to the data whereas others could be considered more abstract from the data. This is because the identification and review of themes occurred at both semantic and latent level [22]. The former was in order not to lose the richness of the raw data reflecting peoples narrated experiences and the latter was to help draw out implicit hermeneutics and underlying patterns and structures of belief as contained in the narrations of the participants.

### Ethics

Ethical approval for this study was obtained from the Ethical Committee of Ebonyi State University, Abakaliki and the London School of Hygiene and Tropical Medicine. Participation in the study was voluntary and a signed written informed consent sheet was obtained from each participant. Participants were anonymised in the analysis. At the FGD, participants agreed to maintain the confidentiality of every conversation made during the session.

## Results

**Fig 1:**
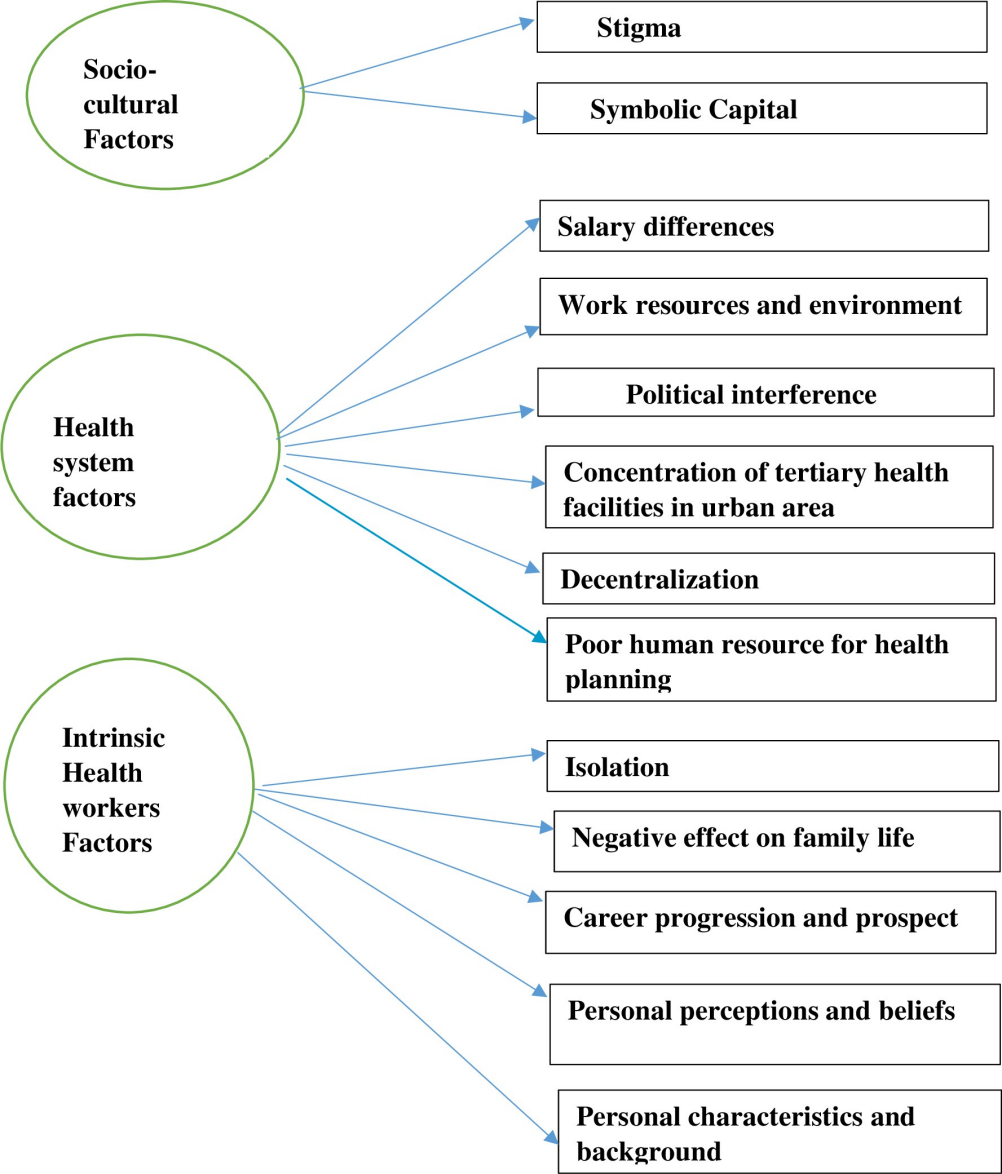
Thematic code for factors affecting the distribution of health workers to rural areas.

The results of our findings can be generally divided into three themes of health system factors, socio-cultural factors and intrinsic health workers factors. Within the first theme, a series of sub-themes emerged: salary differences, work resources and environment, political interference, the concentration of tertiary health facilities in urban areas, decentralization issue and poor human resource for health planning while under the second theme were symbolic capital and stigma,. The sub-themes that emerged for the third theme were Isolation, negative effect on family life, career progression and prospects, personal perceptions and beliefs, personal characteristics and background. This is represented in Fig 1.

### Health system issues and governance

#### Salary differences

This was one of the most alluded issues that made most urban healthcare workers unwilling to work in the rural area, and also responsible for poor retention in these areas. This stems from the issue of a differential salary paid to the disadvantage of the rural workers because they are being paid by different tiers of government. Thus, working in the urban area and to a large extent for the federal government health facilities means you are financially secure as your salary either increases or remains the same with each successive government. This is contrary to the case with the lower tiers of government where it is subject to unpredictable fluctuations depending on the disposition of each new government. Again, the majority of the participants thought that the current rural allowance being paid by all tiers of government was insufficient to counter the foregone benefits of relocating from the urban area. This is illustrated by the quote of this participant:

*"What you earn is more of whom you are working for. . . When a local government employs you as a doctor, what you earn is different from that when the FG employs you."*

(Urban based late-career doctor)

#### Work resources and environment

Some of the recurrent concerns among the HCWs were the poor work environment, and equipment and resources needed to enable them to deliver efficient health services. These appear to be problems in both urban and rural areas but were more severe in rural areas. The buildings and environment were seen to be poorly maintained compared to those in urban areas. Furthermore, the government was seen as being uninterested in providing basic working tools that are necessary to improve the performance of health workers.

*“…So the government pay more attention to facilities in the urban than in the rural because, of course, it is in the urban areas where the policymakers. . .reside so, they pay attention to facilities that serve them more than those ones that serve the commoners, so when you come to facilities in the rural area you may not have the equipment you need to work, the laboratories that support clinical practice are more empty than in the urban area and you don’t have other ancillary staff you know…the paramedics …that will help you in your clinical practice as much as you do in the urban area..”*

(Urban based mid-career to late-career doctor)

#### Political interference

The discussion with the career policymakers revealed that there appears to be a gap in knowledge between them and the appointed political policymakers on what constitutes the provision of good healthcare (health system). They perceived that the appointed ones appear to have wrong ideation of what constitutes providing good healthcare delivery, assuming it to mean the provision of more physical structures like buildings at the expense of adequate staffing and working tools. These misconceptions stem from the drive of the political elites to score political points with the communities and people that elect them. This makes the political appointees to override key suggestions from the career policymakers, only playing according to their political interests as shown by this quote by a career policymaker:

*"*. . .*that is why when they wanted to site another big public hospital here [urban area] less than 1km from the other one*, *I opposed it and said move it to a rural area*, *but they [political decision maker] wouldn’t listen*.*”* (Health policymaker)

#### Skewed distribution of tertiary health facilities in urban areas

This is another concern that arose from the idea that the government situates most tertiary facilities in urban areas which employ a huge number of healthcare workers and specialists, thereby denying the rural areas. These facilities are teaching hospitals that train physicians and nurses at both undergraduate and postgraduate levels. Thus a situation in Abakaliki (the state capital), where two specialist facilities are located less than 1 km from each other was deemed unacceptable. This was illustrated by a comment by a health policymaker

*"*. . .*but they concentrate (everything) in town*. *So*, *location is an issue…"* (Health policymaker)

#### Decentralization

The current separation of responsibility for the funding of healthcare amongst the different tiers of government (with the central government seen as the most financially stable) was identified as a crucial driver for the skewed distribution among the participants. The tier of government responsible for employing rural HCWs was seen as weak and less financially capable. This makes them unable to meet up with the enormous wage bills associated with employing health workers. Furthermore, other perceptions that emerged as a consequence of this separation was that the lower tiers were seen to be more prone to health policy uncertainty (summersault) about the condition of HCWs salaries and benefits, so that policy changes are seen with almost every successive government compared to the more stable central government. This was illustrated by this quote by a participant:

*". . . one of the reasons why I wouldn’t trust working with the state is that a new government can come and overnight they will turn around a whole policy. . . like reduce your salary drastically which doesn’t happen with the FG (central government)"*.

(Urban based mid-career to late-career doctor).

#### Poor human resource for health policy and planning

From the interaction with the policymakers, there is a poor uncoordinated HRH policy and planning in the state. This has currently led to the current state of a "crisis" in healthcare distribution in the rural areas. This is an issue that has persisted over a long time as the government has not systematically had a good HRH plan as illustrated by the fact that they had not conducted a formal employment process for over a decade, spanning two successive governments, despite having a large number of retiring health workers. This has inevitably led to almost a total collapse of the health system as illustrated by this statement by a policymaker:

*"…No*, *No*, *we are just floating*, *there are no junior anything*. *They are not employing new people*, *so that’s the problem*. *And they blame that on the poor economy*. . . *we are top-heavy*.” (Health policymaker)

As a result, the state has had a massive reduction of available health staff who have retired without having any replacements.

### Socio-cultural factors

Two main issues arose from our interactions with the different participants which bothered around symbolic capital and stigma.

#### Symbolic capital

This was a sub-theme that emerged while discussing with the participants. It expresses how little value and recognition the society and culture place on health workers working in rural areas compared to their urban counterparts. Here, participants described how working in rural areas was seen to be un-dignifying and belittling; hence, a less prestigious work. They described how working in rural areas was seen as ’less acceptable’ and was perceived to be the last resort when one was not offered a job in an urban area. This narrative is further reinforced by the preferential treatment and recognition that governments, organisations and society give to workers in urban areas compared to workers in rural areas. This was demonstrated in a comment made by one of the interviewees:

*"Who are the people who are given national honours in Nigeria? They are all the people who live in the urban area, it has never been considered [that] because you sacrificed to work in the rural area [to award you] even the least honour in the land"*

(Urban based mid to late-career doctor)

Contributory to this issue was the perception that the tiers of government (state/local government) that employ rural HCWs were seen to be less powerful and less prestigious compared to the federal or central government that employs the majority of the urban-based HCWs.

#### Stigma

This sub-theme is closely associated with the aforementioned sub-theme on symbolic capital. Here, participants described how working in rural areas is viewed by both society and urban colleagues as devaluing. This also manifests as a form of discrimination as working in rural areas was considered as a work meant for less intelligent individuals who are seen as impoverished. This is one of the quotes from one of the early career health workers:

“*Yes now*, *yes*. *I’ve said it before*, *in the rural setting they look down on the people working in the rural area than people working in the town*. *They think that people in the rural are less ehmm*. . .*intelligent than those in the town”*

(Rural based mid to late-career doctor)

### Intrinsic healthcare worker factors

#### Isolation

There was a feeling that the way workers are posted and their inadequate number lead to isolation. This isolation was reinforced by the fact that in these locations there were few available social networks to be part of (with members of the local community). This narrative also included the concern that they are often removed from their extended family and former social networks, such as friends. Many considered this factor important because there is great value placed on social networks in Nigeria. This is as shown by this quote:

*“In urban areas you meet with a lot of highly talented people. You know, people migrate from the rural area to the urban area just because of this white-collar job, and there you meet calibers of people that will change your 1Q. That will make you to think in another..a higher level, and that will change your perspective as well. Unlike in the rural areas, you are always meeting with tho…se….as I explained to you the elderly, the farmers, lower socio-economic class of people] that’s the people you are concerned with. . . that’s the people you are meeting with.”*

*(*Rural-based early-career nurse)

#### Negative effect on family life

For most healthcare workers, working in a rural area was almost incompatible with their family life aspirations. This stems from working and residing in such areas not offering a conducive environment for raising their children or giving them the quality of schooling they would want for them. Furthermore, it is seen to potentially impact the quality of their family life as there are fewer and less paying employment opportunities for their spouses, absence of other social amenities for recreation, etc.

#### Career progression and prospect

For every one interviewed, this was one of the major reasons that made them not want to work in the rural area, and almost in all cases the reason why they relocated to the urban area. This stems from the fact that the majority of specialist or enhanced medical training institutions (mainly the teaching hospitals) are located in the urban area, with a higher concentration of trainers and more senior colleagues in the urban area. This makes for little or no supervision and lack of adequate mentorship for those that work in rural areas, in addition to reduced peer-based motivation often necessary to strive for better career training and progression. This is illustrated by this quote by a rural physician who has worked for over a decade in a rural area:

*"So I lacked mentorship or supervision and that was what had so much dealt with me…"*

(Rural based mid to late-career doctor)

#### Personal characteristics and background

This was one of the themes that were deduced that could influence some of the healthcare workers to work in rural areas with those who have resided in rural areas in their younger days or having had a prior extensive exposure to rural communities being perceived to be more prone to accepting to work in such areas. It appears that their experiences may make them more accepting of residing and working in such areas. The following quote from a nurse illustrates this point:

*“Because I have done my research and I have found out that most people that work [in a rural area], will tell you that they lived with their grandmother [in a rural area] for so, so period, so they have tasted the life of a rural area so they know and they adapt with rural whatever. Unlike people that grew up all their lives in urban areas they cannot adapt here,”*

(Rural-based early-career nurse)

## Discussion

The findings of this work add to the body of knowledge on the factors that are perceived to be linked to the distribution of HCWs to rural areas, especially in Ebonyi State, and Nigeria as a whole. Furthermore, it has revealed several factors responsible for these such as socio-cultural factors, health system and governance issues, as well as intrinsic HCWs’ factors. The socio-cultural factors mentioned include symbolic capital and stigma while health system and governance issues mentioned include a poor human resource for health policy and planning, work resources and environment, salary differences, decentralization, the concentration of tertiary health facilities in urban area and political interference. The intrinsic healthcare workers’ factors mentioned include career progression and prospect, negative effect on family life, personal characteristics and background isolation, personal perceptions and beliefs.

Our studies revealed some key socio-cultural factors which may serve strongly to inhibit urban healthcare workers moving to rural areas and rather stay in urban areas even in the presence of other pull factors (financial and non-financial incentives). Symbolic capital which has to do with the recognition and prestige accorded to one due to his profession or achievement or position seems to be absent for rural HCWs compared to their urban counterparts. A demonstration of this is the constant overlooking of rural health care providers in the award of honours whether by the government (state or national) and even by their professional unions. This finding is in keeping with the results of a study done in Peru exploring similar issues, which pointed out that the healthcare workers considered public recognition an important incentive and issue to attract and retain health workers in remote and rural areas [29]. The recognition which forms part of the symbolic capital has been identified by the world health organisation as one of the key factors that can influence the performance and productivity of health workers [2, 3]. This finding may not be different from the study in Sierra Leone where health workers in the rural area felt a sense of poor support from the ministry of health which may make them appear to stand alone and essentially unrecognized [30].

Closely related with symbolic capital was stigma, which is linked with working in a rural area due to the perception from the society of not being intelligent hence probably one of the reasons a healthcare worker will be in a rural area and not in the urban area. This finding is crucial considering that rural areas need to attract early career HCWs who may be more affected by the opinions of their peers and society. The issue of stigma may not be surprising given also that people residing in rural areas are not as empowered as those who reside in urban areas with lower economic capital and less prestige. Again the likelihood that a significant proportion of people affected by some of the stigmatizing diseases of poverty such as tuberculosis, leprosy, neglected tropical diseases, etc. is higher. This may have an associated spillover effect as has been seen in other studies which have shown that stigma of health workers can occur if their work makes them associated with people with characteristics or health conditions that society finds less desirable [31].

The negative impact of decentralization as a factor that has driven the current issue is prominent. This is because Nigeria currently runs a three-tier healthcare provision system, with the lowest rung and the least funded being primarily responsible for healthcare service provision and delivery to rural areas, and with poor support from the other two tiers of government [7, 25, 32]. This has caused a clear disparity in salary between HCWs working in the urban area and those working in the rural areas, with workers in the rural area receiving far less. The effect of the disparity in salary in discouraging health workers’ motivation or attraction to work in rural areas has also been highlighted by another study from Tanzania where non-uniform financial incentives were identified as a potential driver for the out-migration of physicians from one district to higher-paying districts [33]. Again, these facilities managed by the lower tiers of government are more prone to policy summersault with each successive government. This has engendered a loss of trust and the undesirability of working at such levels. In Ebonyi State, and to large extent the entire country, healthcare workers prefer to work in the urban areas where facilities are owned more by the highest tier of government (the federal government), unlike the other two tiers. This issue derives from the presence of a much more unstable policy environment, especially as regards the welfare of the workers in the other tiers of government, unlike the federal government. Our findings are similar to earlier report which showed that decentralization is a major reason why people don’t want to work in rural areas [19]. This places the provision and payment of the healthcare provider for rural dwellers largely on the weakest tier of government with the responsibility of with minimal support from the state and the federal government. As shown in other studies, this issue is a major drawback on other pull factors that may be put in place [34]. This stems from the fact that it has other untoward (spillover) effect as most health care providers do not have confidence in the other tiers of government as they are seen to be prone to policy somersault compared to the federal (central) government.

Poor human resource for health policy and planning for the state was another issue that was raised which has manifested in the fact that both the Local and State Governments has not employed any health worker for some time. This is worsened by the fact that there is high attrition of HCWs due to retirement from the service. Preliminary unpublished data showed a higher concentration of healthcare workers in the urban as against the rural area with anecdotal evidence of a large number of under-employed or unemployed healthcare workers who are based in urban areas and may be willing to work in rural areas if the right conditions are provided. The role policy issues can play in the attraction and retention of HCWs in rural areas has also been highlighted by a study in Bangladesh. The study pointed out that the non-implementation of expected policy on retentions of health workers to a rural area is one of the drawbacks for providers’ reluctance to work in rural and remote areas [33].

This work revealed that there are contentions (frictions) between the political class who primarily are the ’policy elites’ responsible for final decisions regarding the acceptance and implementation of policies. There are two forms of health policymakers found in the state who are responsible for the formulation and implementation of health policies in the state. The career policymakers who are civil servants of the ministry of health and the appointed policymakers who are politicians (policy elites) who make the final decision on any policy issue in consultation with the career ones. These appointed policymakers are prone to change with successive governments, unlike the career ones. At times, there exist substantial policy interest gaps between the career policymakers and the political ones on the ideation of what constitutes the provision of quality healthcare. The political policymakers are maybe more concerned about issues that give them physical visibility and adds to their political capital such as the building of more hospitals and health centers compared to solutions such as adequate staffing and provision of working tools/resources that give less visibility. This finding supports earlier results by Onoka and colleagues which revealed that one key issue that resulted in the failure of adoption of the health insurance program in one state in Nigeria compared to their counterpart was the non-support of key political actors compared to the career policymakers [35].

Deliberate policy of accordance of recognition by political actors and other policymakers can go a long way both in improving the value of being a worker in the rural area. This can go along with implementing other measures to enhance the status of health workers who work in such areas which will serve as both a motivating factor in encouraging more health workers to seek to work in such places. This finding is important for a country such as Nigeria which now implements a national health law providing more funding to the health system from the central government to the primary health care levels through the basic health care provision fund [36]. Some portion of the funding targeting human resource issues must look at implementing some of the less financially demanding challenges such as the institution of recognition scheme amongst others.

From these findings it is clear that solving the mal-distribution of human resource for health to rural areas cannot be dealt in isolation as the underlying factors responsible for it are multi-dimensional and interconnected. This study has shown that there are non-financial incentives that are cost-effective and not capital intensive which governments and their partners can employ to encourage more health workers to decide to work in rural areas and even for those already there not to quit. This is in keeping with the WHO recommendations and findings from other authors for the implementation of context-specific strategies in enhancing the attraction and retention of health workers to rural and remote areas in different countries [2, 37]. This presents study highlights the role that stigma and symbolic capital may play in the distribution of health workers which is important in exploring factors influencing the decision to migrate out of or into rural areas using the ‘the pull, push, stick and stay factors’. Lessons from this study clearly demonstrates the need to explore more the implementation of such non-financial schemes like public recognition in a bid to increase the symbolic capital of health workers in rural areas. These may be important in helping to ameliorate stigmatizing attitudes towards health workers in area it may be found as well.

### Strength and limitation

The researchers have strong contextual understanding having had a long-standing working relationship in the environment as both healthcare workers and researchers. This is crucial especially in giving an interpretation to some of the contextual issues the participants brought up during the course of the work and also in interpreting/analysis of the work. The work has some other strengths arising from our use of a qualitative method of inquiry, studying of urban HCWs and policymakers who have prior experience working and residing in rural areas as well as some rural HCWs who previously were working in urban areas. A limitation of the study is the generalizability of the findings given the limited number of sample although qualitative methods are concerned more with understanding the phenomenon than for generalizability.

### Future studies

There is a need for more studies to explore the role symbolic capital can play in the attraction and retention of HCWs from other different contexts as this is not highly visible in most studies. There may also be a need to carry out retrospective policy analysis to understand how agenda setting occurs in the state for its use in attempting to implement the policy changes with this issue. There would be a need to do a more detailed HCWs labour market analysis and a discrete choice experiment incorporating some of these findings including symbolic capital to strengthen the key findings of this work and also as a tool for planning for the human resource for health in the state.

### Conclusion

In summary, this study provides more information on factors that are amenable to policies that are implementable to help attract HCWs to rural areas. One area of concern raised is the role of symbolic capital such as recognition and stigmatized position of being a rural health worker can play. There is the need to set up plans for higher recognition programs or events that will enhance the reputation and prestige of such health workers found in rural areas. Our findings has great implications for policy and practices especially in similar settings where there is the inequitable distribution of HCWs between regions. Our work has shown that there are financial and non-financial incentives that may help attract urban HCWs to rural areas. In keeping with earlier studies, it may be important that government and their partners realize that there may be a need for the consideration of both financial and non-financial incentives in seeking for solutions to ameliorate this issue especially less costly incentives such as the implementation of better recognition scheme [2, 17, 23, 38–41].

## Supporting information

S1 File File containing interview guides. (PDF)

S2 File File containing focus group discussion topic guide. (PDF)
