# Supplementary material for: A qualitative inquiry of rural-urban inequalities in the distribution and retention of healthcare workers in southern Nigeria
Source: PLoS One. 2022 Mar 29;17(3):e0266159. doi: 10.1371/journal.pone.0266159 (PMC8963562; doi:10.1371/journal.pone.0266159)
Supplement: S1 File — (PDF) [file pone.0266159.s001.pdf]

Interview Guide for Healthcare Workers (Urban and Rural) and Health Policymakers.

Study: Factors Influencing the Distribution of Healthcare Workers to Rural areas in Ebonyi state, South-East Nigeria

1. Interview Guide (Urban Healthcare Worker)

Introduction

Thank you very much for allowing me to fix this appointment with you. This project is being carried out as part of my Master's dissertation project and I am interested in understanding the factors that you think about when you decide where you want to work as a health professional. I also seek to understand the factors that are responsible for the current distribution of health workers (Doctors and Nurses) between the rural and urban areas in the state. Finally I am interested in knowing what can help attract more health workers (Doctors and Nurses) to rural areas .This interview should take approximately 45 mins to 1 hour. Your participation will help in informing policies both locally and nationally on how to attract more health workers to rural area by Government and private health providers.

[Have each person read Consent form and sign]

I will now ask you some questions. Please let me know if anything is unclear or confusing.

General Introductory question

1. Can you please introduce yourself?
2. Would you describe your hometown as a township or rural area and have you ever lived in a rural area)

Main content

1. What does the term ‘rural area’ mean to you?
2. What are some of the important issues you consider when thinking about where you want to work?

   (Probe: the scope of practice, the quality (e.g., cleanliness, equipment, reliable drug supply, etc.) of health facilities, safety, support from senior colleagues, management support, MOH support, difference in salary, living conditions(housing ,Communications e.g. phone, Internet, etc., water, electricity, access to social activities)transport and access, Children schooling, opportunities for career promotion or in-service training ,opportunities for study leave ,community support and satisfaction.

3. What in your opinion among the issues could have majorly contributed to your decision not to work in a rural area?
4. Are there differences between working in an urban area and a rural area?

   (Probe: the scope of practice, the quality (e.g., cleanliness, equipment, reliable drug supply, etc.) of health facilities, safety, support from senior colleagues, management support, MOH support, difference in salary, living conditions(housing ,Communications e.g. phone, Internet, etc., water, electricity, access to social activities)transport and access, Children schooling, opportunities for career promotion or in-service training ,opportunities for study leave ,community support and satisfaction.

5. What would it take to make you decide to work in rural area?
6. What do you think can be done (incentives) to attract more doctors or nurses to work in a rural area? If possible by what difference/amount

   (Probe: salary differentials, bonuses, accelerated career growth and opportunities, children’s education allowance, In-service training, differential study leave or residency training, any others).

7. Are there any other factors, which have not yet been mentioned, that are important to you when deciding where you will work?

THANK YOU

2. **Interview Guide (Rural Healthcare Worker)**

Introduction

Thank you very much for allowing me to fix this appointment with you. This project is being carried out as part of my Master’s dissertation project and I am interested in understanding the factors that you think about when you decide where you want to work as a health professional. I also seek to understand the factors that are responsible for the current distribution of health workers (Doctors and Nurses) between the rural and urban areas in the state. Finally I am interested in knowing what can help attract more health workers (Doctors and Nurses) to rural areas .This interview should take approximately 45 mins to 1 hour. Your participation will help in informing policies both locally and nationally on how to attract more health workers to rural area by Government and private health providers.

[HAVE EACH PERSON READ CONSENT FORM AND SIGN]

I will now ask you some questions. Please let me know if anything is unclear or confusing.

General Introductory question

1. Can you please introduce yourself?
2. Would you describe your hometown as a township or rural area and have you ever lived in a rural area)

Main content

3. What does the term ‘rural area’ mean to you?
4. What has motivated you to work in a rural area?
5. What has been your experience working in rural area so far?
6. What are some of the important issues you consider when thinking about where you want to work?(both in terms of the practice, facility and location)

(Probe: the scope of practice, the quality (e.g., cleanliness, equipment, reliable drug supply, etc.) of health facilities, safety, support from senior colleagues, management support, MOH support, difference in salary, living conditions(housing ,Communications e.g. phone,

Internet, etc., water, electricity, access to social activities)transport and access, Children schooling, opportunities for career promotion or in-service training ,opportunities for study leave ,community support and satisfaction.

7. What in your opinion among the issues could have contributed to your decision to work in a rural area?
8. Are there differences between working in an urban area and a rural area?

(Probe: the scope of practice, the quality (e.g., cleanliness, equipment, reliable drug supply, etc.) of health facilities, safety, support from senior colleagues, management support, MOH support, difference in salary, living conditions(housing ,Communications e.g. phone, Internet, etc., water, electricity, access to social activities)transport and access, Children schooling, opportunities for career promotion or in-service training ,opportunities for study leave ,community support and satisfaction.

9. What would it take to make you decide to leave working in a rural area?
10. What do you think discourages more doctors or nurses from working in a rural area?
11. What do you think can be done (incentives) to attract more doctors or nurses to work in a rural area? If possible quantify or describe (Probe: salary differentials, bonuses, accelerated career growth and opportunities, children's education allowance, In-service training, differential study leave or residency training, any others).
12. Are there any other factors, which have not yet been mentioned, that are important to you when deciding where you will work?

THANK YOU

3. **Interview Guide (Health Policy Makers)**

Introduction

Thank you very much for allowing me to fix this appointment with you. This project is being carried out as part of my Master's dissertation project and I am interested in understanding the factors that you think about when you decide where you want to work as a health professional. I also seek to understand the factors that are responsible for the current distribution of health workers (Doctors and Nurses) between the rural and urban areas in the state. Finally I am interested in knowing what can help attract more health workers (Doctors and Nurses) to rural areas .This interview should take approximately 45 mins to 1 hour. Your participation will help in informing policies both locally and nationally on how to attract more health workers to rural area by Government and private health providers.

[Have each person read Consent form and sign]

I will now ask you some questions. Please let me know if anything is unclear or confusing.

General Introductory question

1. Can you please introduce yourself?

Main content

2. How does the ministry term a place as rural and others as urban in terms of their classification?

3. Are there issues with the distribution of health care workers in the state in terms of doctors and nurses in the rural as against urban areas? If Yes, why do you say so? if No-why do you say so?

   (If the answer in 2 was no, discuss the statistics that showed the mal-distribution of doctors and nurses more skewed to the urban area)

4. From your experience what could have contributed to the mal-distribution of Healthworkers more to the urban area? (Probe: MOH policies and support ,health system governance issues,the scope of practice, the quality (e.g., cleanliness, equipment, reliable drug supply, etc.) of health facilities, safety, support from senior colleagues, management support, difference in salary, living conditions(housing

,Communications e.g. phone, Internet, etc., water, electricity, access to social activities)transport and access, Children schooling, opportunities for career promotion or in-service training ,opportunities for study leave ,community support and satisfaction.

5. Based on your experience what is your suggestions on how to change the situation?

(Probe: salary differentials, bonuses, accelerated career growth and opportunities, children’s education allowance, In-service training, differential study leave or residency training, any others).

THANK YOU
