# Supplementary material for: A qualitative inquiry of rural-urban inequalities in the distribution and retention of healthcare workers in southern Nigeria
Source: PLoS One. 2022 Mar 29;17(3):e0266159. doi: 10.1371/journal.pone.0266159 (PMC8963562; doi:10.1371/journal.pone.0266159)
Supplement: S2 File — (PDF) [file pone.0266159.s002.pdf]

Focus group discussion guide

Study: Factors Influencing the Distribution of Healthcare Workers to Rural areas in Ebonyi state, South-East Nigeria

Introduction

Thank you very much for coming today. This project is being carried out as part of my Master's dissertation project and I am interested in understanding the factors that you think about when you decide where you want to work as a health professional. I also seek to understand the factors that are responsible for the current distribution of health workers (Doctors and Nurses) between the rural and urban areas in the state. Finally I am interested in knowing what can help attract more health workers (Doctors or Nurses and midwives) to rural areas .This focus group should take approximately 1-1.5 hours. Your participation will help in informing policies both locally and nationally on how to attract more health workers to rural area by Government and private health providers.

[Have each person read the consent form and sign. Explain how the FGDs will run and that it is informal and everyone should feel free to make their contribution]

I will now ask us some questions. Please let me know if anything is unclear or confusing.

General Introductory question

1. Can you please introduce yourself?

(Please also tell us if you are from a township or rural area and if you have ever worked or lived in a rural area)

Main content

2. What does the term 'rural area' mean to us?
3. What are some of the important issues we consider when thinking about where we want to work?

(Probe: the scope of practice, the quality (e.g., cleanliness, equipment, reliable drug supply, etc.) of health facilities, safety, support from senior colleagues, management support, MOH support, difference in salary, living conditions(housing ,Communications e.g. phone, Internet, etc., water, electricity, access to social

activities)transport and access, Children schooling, opportunities for career promotion or in-service training ,opportunities for study leave ,community support and satisfaction.

4. What in our opinion among the issues could have majorly contributed to our decision not to work in a rural area?

5. Are there differences between working in an urban area and a rural area?

   (Probe: the scope of practice, the quality (e.g., cleanliness, equipment, reliable drug supply, etc.) of health facilities, safety, support from senior colleagues, management support, MOH support, difference in salary, living conditions(housing ,Communications e.g. phone, Internet, etc., water, electricity, access to social activities)transport and access, Children schooling, opportunities for career promotion or in-service training ,opportunities for study leave ,community support and satisfaction.

6. What would it take to make us decide to work in rural area?
7. What do you think can be done (incentives) to attract more doctors or nurses to work in a rural area? If possible by what difference/amount

   (Probe: salary differentials, bonuses, accelerated career growth and opportunities, children’s education allowance, In-service training, differential study leave or residency training, any others).
8. Are there any other factors, which have not yet been mentioned, that are important to us when deciding where we will work?

THANK YOU
